# Stigmatization is common in patients with non-alcoholic fatty liver disease and correlates with quality of life

**DOI:** 10.1371/journal.pone.0265153

**Published:** 2022-04-06

**Authors:** Marta Carol, Martina Pérez-Guasch, Elsa Solà, Marta Cervera, Sara Martínez, Adrià Juanola, Ann T. Ma, Emma Avitabile, Laura Napoleone, Elisa Pose, Isabel Graupera, Maria Honrubia, Marko Korenjak, Ferran Torres, Pere Ginès, Núria Fabrellas, for the LiverHope Consortium Investigators

**Affiliations:** 1 Liver Unit, Hospital Clínic de Barcelona, University of Barcelona, Barcelona, Catalonia, Spain; 2 Institut d’Investigacions Biomèdiques August Pi i Sunyer (IDIBAPS), Barcelona, Catalonia, Spain; 3 Centro de Investigación Biomédica en Red de Enfermedades Hepáticas y Digestivas (CIBEReHD), Barcelona, Catalonia, Spain; 4 School of Medicine and Health Sciences, University of Barcelona, Barcelona, Catalonia, Spain; 5 European Liver Patients Association (ELPA), Brussels, Belgium; 6 Medical Statistics Core Facility, Institut d’Investigacions Biomèdiques August Pi-Sunyer (IDIBAPS), Hospital Clínic, Barcelona, Catalonia, Spain; 7 Biostatistics Unit, Faculty of Medicine, Universitat Autónoma de Barcelona, Barcelona, Catalonia, Spain; School of Digestive & Liver Diseases, Institute of Post Graduate Medical Education & Research, INDIA

## Abstract

**Background and aims:**

Stigmatization is a well-documented problem of some diseases. Perceived stigma is common in alcohol-related liver disease and hepatitis C, but little information exists on stigma in patients with non-alcoholic fatty liver disease (NAFLD). Aim of the study was to investigate frequency and characteristics of perceived stigma among patients with NAFLD.

**Methods:**

One-hundred and ninety-seven patients seen at the liver clinic were included: a study group of 144 patients with NAFLD, 50 with cirrhosis (34 compensated, 16 decompensated), and a control group of 53 patients with alcohol-related cirrhosis. Demographic, clinical, and laboratory data were collected. Quality-of-life was assessed by chronic liver disease questionnaire (CLDQ). Perceived stigma was assessed using a specific questionnaire for patients with liver diseases categorized in 4 domains: stereotypes, discrimination, shame, and social isolation.

**Results:**

Perceived stigma was common in patients with NAFLD (99 patients, 69%) and affected all 4 domains assessed. The frequency was slightly higher, yet not significant, in patients with NAFLD cirrhosis vs those without (72% vs 67%, respectively; p = 0.576). In patients without cirrhosis perceived stigma was unrelated to stage of disease, since frequency was similar in patients with no or mild fibrosis compared to those with moderate/severe fibrosis (66% vs 68%, respectively). There were no differences in perceived stigma between patients with compensated cirrhosis and these with decompensated cirrhosis. Among patients with cirrhosis, stigmatization was more common in alcohol-related vs NAFLD-cirrhosis, yet differences were only significant in two domains. In patients with NAFLD, perceived stigma correlated with poor quality-of-life, but not with demographic or clinical variables.

**Conclusions:**

Perceived stigmatization is common among patients with NAFLD independently of disease stage, is associated with impaired quality-of-life, and may be responsible for stereotypes, discrimination, shame, and social isolation, which may affect human and social rights of affected patients.

## Introduction and background

Liver diseases are frequently stigmatized possibly because of their association with alcoholism and drug abuse. In fact, studies have shown that patients with hepatitis C and B infection are frequently stigmatized regardless of the method of transmission of the virus [1–4]. Likewise, patients with cirrhosis are frequently stigmatized irrespective of the etiology of the disease [5, 6].

Stigma was first defined as the situation of the individual who is disqualified from full social acceptance with an obvious consequence of social isolation [7]. A more recent definition characterizes stigma as a social process, experienced or anticipated, characterized by exclusion, rejection, blame or devaluation that results from experience, perception or reasonable anticipation of an adverse social judgment about a person or a group [8]. Unfortunately, a large number of diseases suffer from stigmatization, including AIDS, neurological disorders such as multiple sclerosis and epilepsy, obesity, and a number of chronic diseases, including liver diseases [5, 6, 9–15]. Stigmatization has a large array of potential consequences on patients suffering from these diseases that include depressive symptoms, feelings of helplessness and isolation, shame, poor psychological function, decreased physical and social activity, poor self-image, and reduced quality of life [1, 4, 10, 11, 16–20]. Stigmatization is currently considered a very relevant issue in some diseases because of its potential negative impact on patient health status and the fact that it reduces the possibility of patient access to care and recovery. In recent years, there has been a growing interest on fighting stigmatization of certain diseases with the objective of improving patients’ social acceptance and overall health status. Some of these initiatives involve several stakeholders, including patients associations, scientific associations, healthcare professionals, and policymakers [21].

Non-alcoholic fatty liver disease (NAFLD) is the most common liver disease worldwide that affects approximately 24% of the world population and is frequently associated with obesity and metabolic syndrome [22]. NAFLD is a progressive disease and a significant proportion of patients evolve from simple steatosis to cirrhosis or hepatocellular carcinoma during follow-up. It is possible that NAFLD is a stigmatized disease because it affects the liver and is frequently associated with obesity, two conditions that are frequent drivers of stigma. However, despite of this and the growing prevalence and increasing clinical interest in the disease, the information on stigmatization in patients with NAFLD is very scant. In fact, stigma of NAFLD is not mentioned in major guidelines of international societies [23, 24]. The most important mentions of stigma come from patients experiences posted in websites of organizations on liver diseases [25–29]. Along these lines, the European Liver Patients Association (ELPA) has brought attention to stigma as a significant problem in patients with NAFLD in some of its recent activities [30–36].

In this context, we designed a prospective study to evaluate the frequency and characteristics of perceived stigmatization in patients with NAFLD. Since NAFLD is frequently associated with impaired quality of life (QOL), we also explored the potential relationship between perceived stigma and QOL in these patients. Our findings indicate that perceived stigma is common in patients with NAFLD and is associated with impaired QOL.

## Patients and methods

### Study objective

The main objective of the study was to investigate the frequency and characteristics of perceived stigmatization in patients with NAFLD. Secondary objectives were to investigate differences in perceived stigma between patients in different stages of NAFLD (without cirrhosis, with compensated cirrhosis and with decompensated cirrhosis), compare perceived stigma in patients with NAFLD-cirrhosis with that of patients with alcohol-related cirrhosis, and, finally, assess whether perceived stigma correlates with patients QOL, as assessed by chronic liver disease questionnaire (CLDQ).

### Patient population

Patients with the diagnosis of NAFLD seen at the clinics of chronic liver diseases and NAFLD of the Liver Unit of the Hospital Clínic of Barcelona were screened as potential candidates for inclusion in the study. The diagnosis of NAFLD required: 1/ abnormal liver tests and/or abnormal liver ultrasonography suggestive of steatosis; 2/ exclusion of alternate etiologies of chronic liver disease, including chronic viral hepatitis, autoimmune liver disease, primary biliary cholangitis, obstruction of biliary tree, drug liver toxicity, hemochromatosis, Wilson’s disease, and alpha-1-antitrypsin deficiency; 3/ history of alcohol consumption of less than 21 units/week in males and 14 units/week in females; and 4/ histological diagnosis compatible with NAFLD in patients in whom liver biopsy was performed [23, 24]. Exclusion criteria were: 1/ age younger than 18 years; 2/ diagnosis of NAFLD made less than 12 months prior to the inclusion in the study; 3/ associated HIV infection; 4/ previous organ transplantation; 5/ important linguistic barrier; and 6/ lack of informed consent. Patients with a very recent diagnosis of NAFLD were excluded from the study because it was considered that they probably did not have enough time with diagnosis of liver disease to perceive the effects of stigmatization. Patients meeting the inclusion criteria without exclusion criteria were invited to participate in the study after explaining its characteristics and objectives. Patients with alcohol-related cirrhosis were also screened for inclusion in a comparative control group. All patients were identified from the electronic medical record system and were contacted by phone to explain them the characteristics and objectives of the study. Patients interested in participating were seen the day of the consultation to further explain the objectives of the study, sign the informed consent, and fill in all the information requested as well as the questionnaires. Patients were invited to fill in the questionnaires, alone, without family relatives, and with no time restriction. Patients with descompensated cirrhosis were invited to participate at least one month after an episode of acute descompensation with or without hospitalization. During the study period, 463 patients with chronic liver diseases were screened and 266 were excluded for different reasons (main reasons for exclusion were diagnosis made within less than 1 year in 159 cases, cognitive dysfunction in 27, and lack of informed consent in 35); therefore 197 patients constitute the study population, 144 with NAFLD and 53 with alcohol-related cirrhosis. The study was approved by the Ethics Committee and all patients provided written informed consent (study reference number HCB 2018/1080).

### Data collection

Demographic and clinical information was collected from the medical records of each patient. Liver tests had been performed within a period of less than 3 months before inclusion in the study in all subjects. Liver stiffness (LS) and control attenuation parameter (CAP) values were also collected if transient elastography had been performed within the previous 12 months before inclusion in the study.

### Chronic liver disease questionnaire (CLDQ)

The CLDQ was administered to all patients included. The CLDQ is a liver disease-specific tool to assess areas of function important to patients with chronic liver diseases [37, 38], which has 29 items contained within 6 domains that include: abdominal symptoms, fatigue, systemic symptoms, activity, emotional function, and worry. The tool uses a Likert scale response format for all items ranging from 1 (most important) to 7 (least important) and has been validated in Spanish version [38]. Scoring of each domain was performed by adding scores for each item and dividing by the total number of items of each domain. Overall CLDQ score was obtained by adding scores for each item and dividing by 29, which is the total number of items.

### Hepatic stigma questionnaire

The questionnaire used for assessment of perceived hepatic stigma was designed by Vaughn-Sandler et al. [5]. This tool was developed in patients with cirrhosis of different etiologies and is based on 18 stigma-related questions that are categorized in 4 domains: stereotypes (questions 1 to 4), discrimination (5 to 7), shame (8 to 11), and social isolation (12 to 18) (see S1 Table). Answers to questions are made on a 4-point Likert scale: “strongly disagree”, “disagree”, “agree” and “strongly agree”. The main analysis was performed using the 4 different domains. A given patient was considered positive for a particular domain if the answer to at least one question of the domain was one of the two highest in Likert scale, either “agree” or “strongly agree”.

### Statistical analysis

Quantitative variables are expressed as mean plus/minus SD or median and IQR. Qualitative variables are expressed in numbers of percentages. Evaluation of significant differences between groups for continuous variables was performed using the Student t test and ANOVA or Kruskal-Wallis where appropriate. Comparisons between frequency data for significant differences were performed with chi-square or Fisher’s exact test, where appropriate. Correlations between quantitative assessment of perceived stigma (overall and its components) and CLDQ (total and its domains) were performed with Pearson coefficient. Cronbach’s alpha coefficient was used to assess the scores’ reliability. Significance was set at the p<0.05 level (two-sided). Statistical analysis was performed using SAS version 9.4 software (SAS Institute Inc., Cary, NC, USA).

### Sample size

A sample size of 198 subjects randomly selected will suffice to estimate with an 84% confidence and a precision +/- 5 percent units, a population percentage considered to be around 50%. Population was divided into three subgroups according to disease stage. Patients with NAFLD without cirrhosis, patients with NAFLD with compensated cirrhosis and patients with NAFLD with decompensated cirrhosis. Additionally, a group of patients with alcohol-related cirrhosis was included as a control group.

## Results

### Characteristics of the patient population

A total of 197 patients were included in the study, 144 with NAFLD and 53 with alcohol-related cirrhosis. Among patients with NAFLD, 94 had different stages of fibrosis but without cirrhosis and 50 had cirrhosis (34 compensated, 16 decompensated). The demographic and clinical characteristics of patients with NAFLD are shown in Table 1. Median age was 64 years and 52% were male. As expected, risk factors for NAFLD were very common, including obesity, type 2 diabetes mellitus, and arterial hypertension. Seventy-six percent of patients had education of high school or college level. In the cirrhosis group the diagnosis was confirmed by liver biopsy in 34 (68%) cases. In the remaining 16 patients evidence for the diagnosis of cirrhosis derived from previous decompensations in 10 patients and biological signs of liver failure together with evidence of portal hypertension in abdominal ultrasonography in 6 patients. In the non-cirrhosis group, the diagnosis was confirmed by liver biopsy in 45 (48%). In the remaining 49 patients liver function tests, including serum bilirubin, serum albumin and prothrombin time, were normal, abdominal ultrasonography did not show signs of portal hypertension, and median liver stiffness was 6.7 kPa (IQR 4.8–11.3). In the overall group, median time elapsed since diagnosis of NAFLD until inclusion in the study was 2.7 years (1.3–5.5).

**Table 1:** Baseline characteristics of patients with NAFLD included in the study categorized according the stage of the disease*.

	NAFLD without cirrhosis (n = 94)	NAFLD compensated cirrhosis (n = 34)	NAFLD decompensated cirrhosis (n = 16)	All patients (n = 144)	P value #
Age (years)	62 (54–69)	69 (60–72)	54 (52–65)	64 (56–70)	**0.033**
Gender (male)	52 (55)	16 (47)	7 (44)	75 (52)	0.556
Marital Status Single/married/divorced/widowed (%)	10/63/12/8**	2/25/4/3	2/10/4/0	14/98/20/11	0.944
Education status Primary school/high school/bachelor’s or master’s degree (%)	16/47/30**	12/13/9	5/6/5	33/66/44	0.265
Diabetes Mellitus (%)	47 (50)	23 (68)	10 (63)	80 (56)	0.176
Arterial hypertension (%)	57 (61)	21 (62)	8 (50)	86 (60)	0.099
Obesity (%)	60 (64)	17 (50)	9 (56)	86 (60)	0.357
BMI (kg/m^2^)	31 (29–35)	30 (28–33)	31 (28–33)	31 (28–34)	0.469
Time since diagnosis (years)	2.2 (1.3–5.4)	3.9 (1.7–6.9)	2.6 (1.1–6.2)	2.7 (1.3–5.5)	0.218
Hepatic Encephalopathy (%)	0 (0)	0 (0)	6 (38)	6 (4)	**<0.001**
Gastrointestinal bleeding (%)	0 (0)	0 (0)	6 (38)	6 (4)	**<0.001**
Ascites (%) 94/48/140	0 (0)	0 (0)	12 (75)	12 (8)	**<0.001**
Bacterial Infections (%) 94/47/139	0 (0)	1 (3)***	1 (6)	2 (1)	**0.009**
AST (IU/L)	32 (23–48)	28 (25–47)	36 (24–44)	32 (24–47)	0.953
ALT (IU/L)	40 (27–61)	31 (26–52)	25 (21–33)	34 (25–56)	**0.012**
GGT (IU/L)	54 (32–89)	46 (31–73)	64 (39–80)	60 (33–89)	0.807
Bilirubin (mg/dL)	0.6 (0.5–0.9)	0.7 (0.5–1)	0.9 (0.8–1.6)	0.7 (0.5–0.9)	**0.008**
Albumin (g/L)	47 (44–48)	46 (43–47)	43 (37–46)	46 (43–48)	**<0.001**
INR	0.98 (0.93–1.1)	1.05 (0.99–1.09)	1.18 (1.08–1.26)	1.0 (1.0–1.1)	**<0.001**
Liver stiffness (kPa) ****	6.5 (4.8–11.4)	16.3 (10.1–21.3)	31.9 (20.3–36.4)	9.3 (5.6–16.3)	**<0.001**
CAP (dB/m) ****	317 (289–353)	289 (240–320)	251 (228–304)	308 (270–341)	**0.001**

* Values are numbers or percentages (in brackets) or medians (IQR).

# Comparison between the 3 groups of patients

** Data available in 93 patients.

*** Data available in 31 patients.

**** Available in 135 patients, 94 without cirrhosis and 41 with cirrhosis, within the previous 12 months before inclusion in the study.

As mentioned before, a group of patients with alcohol-related cirrhosis (n = 53) was included in the study as positive control group. Comparison of characteristics of these patients and those of patients with NAFLD-cirrhosis are shown in S2 Table. More than half of patients with alcohol-related cirrhosis (28, 53%) had the diagnosis confirmed by liver biopsy. Most patients (44, 83%) had been abstinent from alcohol for at least 6 months before the study. There were no significant differences between groups with respect to the severity of cirrhosis, as estimated by liver function tests or severity scores (MELD and Child-Pugh). However, the median time elapsed since diagnosis of cirrhosis until inclusion in the study was significantly longer in patients with alcohol-related compared to NALFD-cirrhosis (6.6 vs 3.3 years, p<0.001) and a higher number of patients with alcohol-related cirrhosis had had previous episodes of acute decompensation, particularly ascites and hepatic encephalopathy, which indicates a longer exposure to the disease together with a more severe course. As expected, obesity, type-2 diabetes and other components of metabolic syndrome were significantly more frequent in patients with NAFLD-cirrhosis than in those with alcohol-related cirrhosis.

### Internal reliability of perceived stigma scale

For the perceived hepatic stigma scale, Cronbach’s α score in the whole series of patients was 0.750 indicating strong internal consistency reliability of the scale in this series. Similar α scores were found in patients with NAFLD, either overall or categorized according to presence or absence of cirrhosis, and in patients with alcohol-related cirrhosis (reliability coefficient values of 0.717, 0.698, 0.728, and 0.732, respectively).

### Perceived stigma in patients with NAFLD

**Fig 1:**
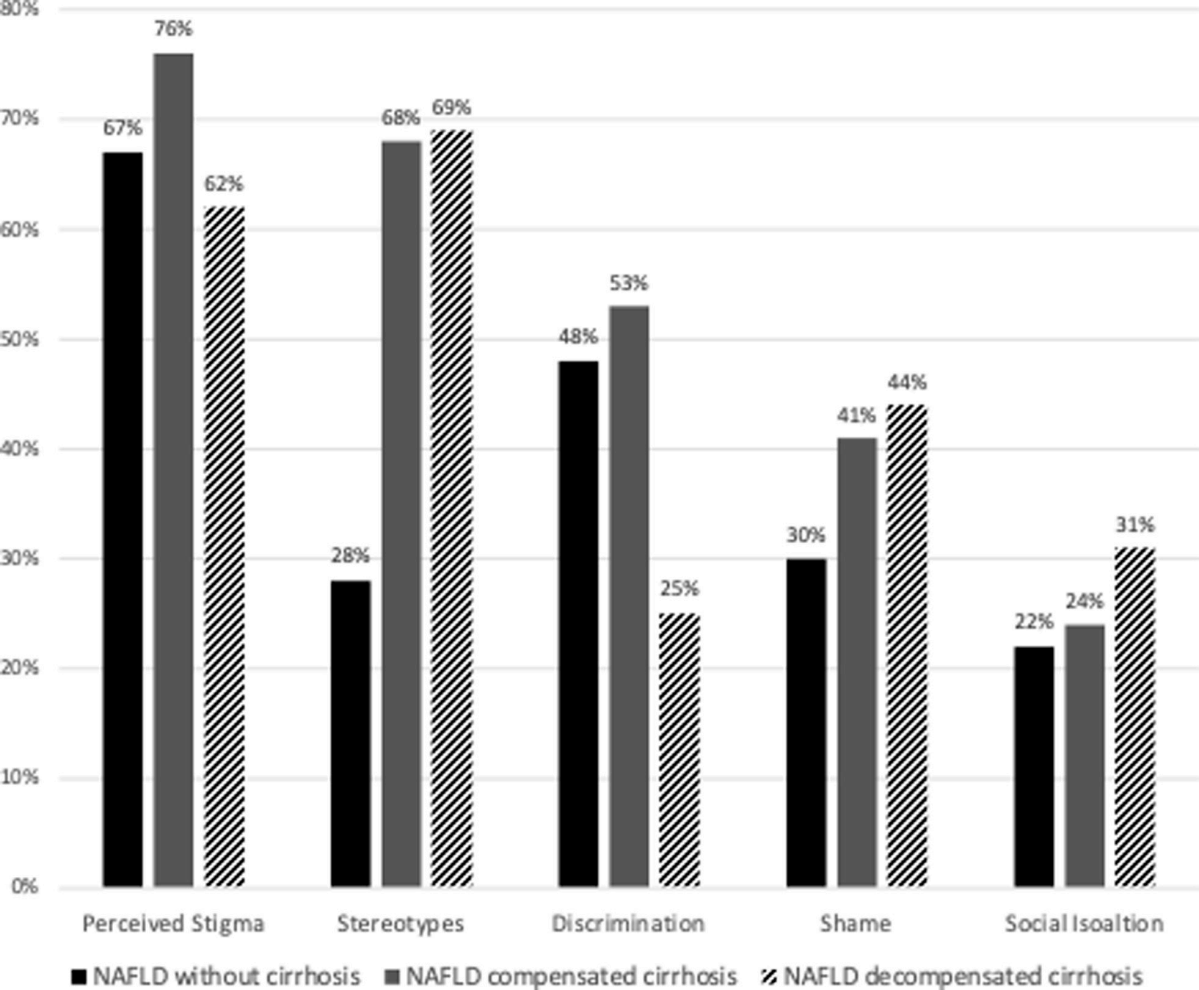
Frequency of perceived stigma, overall and in four different domains, in patients with NAFLD without cirrhosis, NAFLD with compensated cirrhosis compensated and NAFLD with decompensated cirrhosis.

A high proportion of patients with NAFLD indicated they felt stigmatized by providing an affirmative answer (either “agree” or “strongly agree”) for at least one of the stigma-related questions (99 out of the 144 patients, 69%). Frequency of perceived stigma was higher in patients with NAFLD-cirrhosis compared to that of patients without cirrhosis, yet the difference did not reach statistical significance (72% vs 67%, respectively; p = 0.576). There were no significant differences in perceived stigma between patients in different disease stages (Fig 1). Questions that were answered affirmatively by more than 20% of patients belonged mainly to stereotypes, discrimination and shame (Table 2). Questions answered affirmatively by a very low proportion of patients (<5%) are shown in S3 Table. To investigate whether fibrosis severity was related to perceived stigma in patients without cirrhosis, patients were classified into two subgroups using the severity of fibrosis in the liver biopsy or liver stiffness as cutoffs (<F2 or ≥F2 or <10kPa or ≥10kPa, respectively). There were no significant differences in any of the domains of stigma, which suggests that perceived stigma is related to the NAFLD “per se” and not to fibrosis severity (Table 3).

**Table 2:** Stigma-related questions that were answered affirmatively by more than 20% of patients with NAFLD without cirrhosis (panel A), compensated cirrhosis (panel B) and decompensated cirrhosis (panel C), classified according to the different domains.

QUESTIONS	
**A**	
**STEREOTYPES**	
Other people think I am partially to blame for my liver disease.	23 (25%)
**DISCRIMIATION**	
People with liver disease are looked down upon by society	43 (47%)
**SHAME**	
I feel like I am partially to blame for my liver disease	20 (22%)
**B**	
**STEREOTYPES**	
Some people assume that because I have liver disease, I must have been a drinker	9 (26%)
**DISCRIMIATION**	
People with liver disease are looked down upon by society	16 (47%)
**SHAME**	
I feel like I am partially to blame for my liver disease	9 (26%)
I feel less competent that I did before I was diagnosed with liver disease	7 (21%)
**C**	
**STEREOTYPES**	
Other people think I am partially to blame for my liver disease.	5 (33%)
**DISCRIMIATION**	
People with liver disease are looked down upon by society	4 (27%)
**SHAME**	
I feel like I am partially to blame for my liver disease	3 (20%)
I feel less competent that I did before I was diagnosed with liver disease	7 (47%)
**SOCIAL ISOLATION**	
I avoid telling other people about my liver disease	4 (27%)

**Table 3:** Comparison between frequency of affirmative answers to stigma-related questions divided by each domain of patients with NAFLD without cirrhosis classified according to fibrosis severity*.

	No or mild fibrosis (n = 56)	Moderate/severe fibrosis (n = 38)	p
**Total**	37 (66%)	26 (68%)	0.812
**Stereotypes**	15 (27%)	11 (29%)	0.818
**Discrimination**	28 (50%)	17 (45%)	0.616
**Shame**	20 (36%)	9 (24%)	0.215
**Social Isolation**	13 (23%)	8 (21%)	0.805

* No or mild fibrosis: <F2 in liver biopsy or liver stiffness <10kPa. Moderate or severe fibrosis: ≥F2 in liver biopsy or liver stiffness ≥10kPa.

### Quality of life and relationship with perceived stigma

Comparison of scores of different domains of CLDQ between NAFLD patients in different disease stages showed no significant differences, except for an expected lower score in activity domain in patients with decompensated cirrhosis (Table 4).

**Table 4:** Chronic liver diseases questionnaire (CLDQ) scores of the different domains in patients with NAFLD included in the study categorized according the different disease stages*.

	NAFLD without cirrhosis (n = 90)**	NAFLD compensated cirrhosis (n = 34)	NAFLD decompensated cirrhosis (n = 16)	p
**Abdominal symptoms**	6.3 (4.9–7.0)	6.7 (4.3–7)	5.5 (4.8–6.3)	0.155
**Fatigue**	5.7 (4.2–6.6)	5.4 (4.2–6.6)	4.5 (3.9–5.7)	0.223
**Systemic symptoms**	6.0 (4.6–6.4)	5.8 (4.4–6.4)	5.1 (3.9–6.3)	0.442
**Activity**	5.7 (4.7–6.7)	5.7 (3.7–6.3)	4 (3–5.8)	**0.013**
**Emotional function**	5.6 (4.3–6.6)	5.3 (4.3–6.4)	5.1 (3.8–6.8)	0.502
**Worry**	6.0 (5.0–6.8)	6.2 (4.2–7)	5.7 (4–6.4)	0.336
**TOTAL**	5.7 (4.7–6.4)	5.8 (4.6–6.1)	5.1 (4–5.7)	0.078

*Values are medians and IQR (in brackets).

** Available in 90 of 94 patients included.

In the whole group of patients with NAFLD, overall perceived stigma showed a mild correlation with the total CLDQ score (r = -0.263; p = 0.002) and its domains (abdominal symptoms [r = -0.186, p = 0.027], fatigue [r = -0.247, 0.003], systemic symptoms [r = -0.221, p = 0.008], emotional function [r = -0.285, p = 0.001), and worry [r = -0.309, p = 0.001], but not with activity [r = -0.112, p = 0.184]). Perceived stigma and CLDQ and its domains were also correlated in patients with NAFLD without cirrhosis (r = -0.319, p<0.002). No relationship was found between perceived stigma and any of the demographic and clinical variables or the presence or absence of cirrhosis. Table 5 shows the CLDQ scores in patients with NAFLD without cirrhosis categorized according to presence or absence of perceived stigma. Notably patients with perceived stigma showed a more marked impairment of CLDQ in all domains but one, indicative of a poorer QOL.

**Table 5:** Chronic liver diseases questionnaire (CLDQ) scores of the different domains in patients with NAFLD without cirrhosis classified according to presence or absence of perceived stigma*.

	Perceived stigma (n = 59)	Non perceived stigma (n = 31)	p
**Abdominal symptoms**	6 (4.3–7)	7 (5.7–7)	**0.018**
**Fatigue**	5 (3.8–6.4)	6.2 (5.4–6.8)	**0.002**
**Systemic symptoms**	5.9 (4.4–6.4)	6 (5.4–6.8)	**0.016**
**Activity**	5.7 (4.3–6.7)	6 (5.3–7)	0.067
**Emotional function**	5.4 (4–6.3)	6.4 (5–6.9)	**0.004**
**Worry**	5.8 (4.6–6.6)	6.6 (5.8–7)	**0.002**
**TOTAL**	5.4 (4.3–6.3)	6.4 (5.5–6.7)	**0.002**

Values are medians and IQR (in brackets)

* Available in 90 of 94 patients included.

### Comparison of patients with NAFLD-cirrhosis and alcohol-related cirrhosis

To further explore perceived stigma in NAFLD, we compared the group of patients with cirrhosis due to NAFLD with a contemporary group of patients with alcohol-related cirrhosis. As mentioned before, in patients with alcohol-related cirrhosis the period of time since diagnosis of liver disease was longer than that of patients with NAFLD-cirrhosis and they had a greater frequency of acute decompensations, likely indicating a more severe disease status of patients with alcohol-related cirrhosis.

Comparison of CLDQ values between the two groups of patients with cirrhosis showed a significant impairment in abdominal symptoms, activity, and overall score in patients with NAFLD-cirrhosis vs alcohol-related cirrhosis (S4 Table). The frequency of perceived stigmatization was higher in patients with alcohol-related cirrhosis than in those with NAFLD-cirrhosis, (91% vs 72%, respectively, p = 0.015). moreover, the frequency of patients that felt stigmatized in the different domains was significantly higher in patients with alcohol-related cirrhosis for the domains of stereotypes, shame and social isolation, but not for discrimination (Table 6). Only three questions were answered affirmatively by a significantly higher percentage of patients with alcohol-related cirrhosis vs NAFLD-cirrhosis (Table 7).

**Table 6:** Comparison between affirmative answers of stigma-related questions divided by each domain of patients with NAFLD cirrhosis and patients with alcohol-related cirrhosis.

	Alcohol-related Cirrhosis (n = 53)	NAFLD-cirrhosis (n = 50)	p
**Total**	48 (91%)	36 (72%)	**0.015**
**Stereotypes**	42 (79%)	16 (32%)	**<0.001**
**Discrimination**	29 (55%)	22 (44%)	0.277
**Shame**	40 (76%)	21 (42%)	**0.001**
**Social isolation**	24 (45%)	13 (26%)	**0.041**

Values are medians and IQR (in brackets).

**Table 7:** Stigma-related questions that were answered affirmatively by a significantly higher percentage of patients with alcohol-related cirrhosis compared to NAFLD-cirrhosis.

QUESTIONS	Alcohol-related Cirrhosis (n = 53)	NAFLD-cirrhosis (n = 50)	p
Some people assume that because I have liver disease, I must have been a drinker	24 (45%)	9 (18%)	**0.006**
Other people think I am partially to blame for my liver disease.	40 (76%)	11 (22%)	**<0.001**
I feel like I am partially to blame for my liver disease	33 (62%)	12 (24%)	**<0.001**

## Discussion

Findings of the current study demonstrate that the majority (69%) of patients with NAFLD in different stages of the disease perceive some kind of stigmatization in their regular lives. This perceived stigma affects all four domains of the stigma assessed, yet to a different degree, including stereotypes, discrimination, shame, and social isolation. Interestingly, perceived stigma was associated with impairment of quality of life that is common in patients with NAFLD.

Liver diseases are considered as stigmatized diseases, yet the information available is mainly limited to certain etiologies, particularly alcohol-related liver disease and hepatitis B or C infection, likely because these etiologies are linked to substance abuse, alcohol and illicit drugs, respectively [4, 39, 40]. Surprisingly, there is very limited information on the perceived stigma of liver cirrhosis which is the final stage of chronic liver inflammation due to multiple etiologies. To our knowledge only two studies have been reported assessing the prevalence, characteristics, and consequences of stigma in patients with cirrhosis. In one study, Vaughn-Sandler et al. [5] investigated perceived stigma in a series of 149 patients with cirrhosis of different etiologies. The vast majority of patients (89%) felt stigmatized in at least one aspect of their lives and stigma was associated with young age and hepatitis C infection or alcohol as etiologies of cirrhosis. Importantly, stigma had a marked negative effect on patients’ lives, as patients with higher perceived stigma had less social support, were less likely to seek medical care, suffered from more depression, and had worse quality of life. The second study was a qualitative study including 15 patients with cirrhosis and reported that perceived stigma was very common and had a negative impact on patients’ ability to cope with their disease and treatment [41].

Here we report the results of a prospective study specifically designed to assess the prevalence of perceived stigmatization in patients with NAFLD. A group of patients with alcohol-related cirrhosis was also studied for comparison. NAFLD was selected because it is the most frequent liver disease worldwide, it is a progressive disease in some patients that can evolve through different stages from simple fatty liver to varied degrees of fibrosis and finally cirrhosis, and is frequently related with overweight and obesity, two conditions commonly associated with stigmatization [15, 21, 22]. The population studied covers the wide spectrum of patients with NAFLD, from simple fatty liver without fibrosis, to significant liver fibrosis but without cirrhosis, to compensated cirrhosis and, finally, decompensated cirrhosis. Our results show that approximately two thirds of the population studied had perceived stigmatization. There was no unanimity in the domains of stigma assessed; rather the positive answers were distributed among the 4 different domains, stereotypes, discrimination, shame, and social isolation. Statements from the questionnaire that were more frequently mentioned were: 1) people with liver disease are looked down upon the society; 2) I feel less competent that I did before I was diagnosed of liver disease; 3) other people feel I am partially to blame because for my liver disease; 4) I feel I’m partially to blame for my liver disease; and 5) I avoid telling other people about my liver disease. Interestingly, there was no association between prevalence of perceived stigma and age, sex, educational background, demographic data, comorbid conditions, including obesity, presence or absence of cirrhosis. Notably, perceived stigma was similar in patients with decompensated cirrhosis compared to these with compensated cirrhosis. Of note, the prevalence of perceived stigma either in the overall score and in different domains was significantly higher in patients with alcohol-related cirrhosis than that in patients with NAFLD-cirrhosis, except for discrimination domain. Differences may be due to the effect of alcohol abuse and may reflect actual differences between the two conditions; however they may also be related, at least in part, to the fact that patients with alcohol-related cirrhosis included in our study had longer duration of the disease and more frequent decompensations vs those with NAFLD-cirrhosis.

As expected, patients with NAFLD showed impairment in QOL, as assessed by CLDQ. This impaired QOL was of similar intensity to that reported in other series of patients in previous studies and also similar to the control group of patients with alcohol-related cirrhosis, yet impairment in activity score and abdominal symptoms was more marked in patients with NAFLD than in those with alcohol-related cirrhosis [38, 42, 43]. Interestingly, perceived stigma was associated with impaired CLDQ values in the overall score, and more markedly altered values in all domains. These findings strengthen the importance of stigma in patients with NAFLD by linking it with the impairment in QOL. Studies should be performed to evaluate whether measures to fight against stigma may improve the QOL of patients with NAFLD.

The current study has several limitations that should be mentioned. The population studied did not include patients with very advanced decompensated cirrhosis, i.e. patients in the waiting list for liver transplantation. Nonetheless, as mentioned before it included a wide spectrum of the disease from simple fatty liver to cirrhosis with portal hypertension and decompensated cirrhosis. The study was performed in a single center which is a tertiary referral center for liver diseases and findings may be different in patients seen in the community by general practitioners or in less specialized centers. The CLDQ-NAFLD questionnaire which is a specific questionnaire to assess QOL in patients with NAFLD was not used in this study [44]; the standard CLDQ was used instead. The reason for this was that a group of patients with alcohol-related cirrhosis was analyzed as controls and for these patients, the standard CLDQ seemed more appropriate. Finally, the possible effect of stigma on areas other than QOL, such as psychological status or access to care was not investigated. This would require additional specific studies.

In conclusion, the results of the current study represent a warning sign about the importance of perceived stigma in patients with NAFLD, which affects very sensitive areas of life. This stigma can result in discrimination and social isolation, and affect human and social rights and the health of affected patients. Importantly, we found an association between stigma and several domains of the CLDQ, which suggests that stigma may contribute, at least in part, to the impaired QOL of patients with NAFLD. We believe that these findings are important for healthcare workers, patients associations, and policymakers and should stimulate further investigation as well as encourage initiatives aimed at preventing discrimination of subjects affected by NAFLD.

## Supporting information

S1 Table Questionnaire of perceived stigma for patients with liver diseases. (DOC)

S2 Table Comparison of characteristics of patients with alcohol-related cirrhosis and NAFLD-cirrhosis. (DOC)

S3 Table Stigma-related questions that were answered affirmatively by less than 5% of patients with NAFLD all stages, classified according to the different domains. (DOC)

S4 Table Chronic liver diseases questionnaire (CLDQ) scores of the different domains in patients with NAFLD-cirrhosis and patients with alcohol-related cirrhosis. (DOC)

## Abbreviations

NAFLD: Non-alcoholic fatty liver disease
CLDQ: chronic liver disease questionnaire
QOL: quality of life
MELD: model for end-stage liver disease
