# Supplementary material for: Stigmatization is common in patients with non-alcoholic fatty liver disease and correlates with quality of life
Source: PLoS One. 2022 Apr 6;17(4):e0265153. doi: 10.1371/journal.pone.0265153 (PMC8986095; doi:10.1371/journal.pone.0265153)
Supplement: S1 Table — (DOC) [file pone.0265153.s001.doc]

**S1 Table**: Questionnaire of perceived stigma for patients with liver diseases *

| **STEREOTYPES** |
| --- |
| Some people assume that because I have liver disease, I must have been a drinker |
| Other people think I am partially to blame for my liver disease. |
| I feel like some people are concerned that my liver disease could be contagious |
| I feel like other people think I am a bad person because I have liver disease. |
| **DISCRIMIATION** |
| People with liver disease are looked down upon by society |
| Some doctors or nurses don’t like taking care of patients with liver disease |
| I feel I have been treated with less respect by others because of my liver disease |
| **SHAME** |
| I feel like I am partially to blame for my liver disease |
| I feel less competent that I did before I was diagnosed with liver disease |
| Because of my liver disease, I feel flawed and incomplete |
| Because of my liver disease, I sometimes feel useless |
| **SOCIAL ISOLATION** |
| I avoid telling other people about my liver disease |
| I feel lonely more often than usual because of my liver disease. |
| I feel like I am an outsider because of my liver disease |
| I avoid doing some things in public because of my liver disease |
| My liver disease makes me stand out to other people |
| Some people avoid me because of my liver disease |
| I feel abandoned by family members because of my liver disease. |

* from Vaughn-Sandler et al (5).
