# Supplementary material for: Stigmatization is common in patients with non-alcoholic fatty liver disease and correlates with quality of life
Source: PLoS One. 2022 Apr 6;17(4):e0265153. doi: 10.1371/journal.pone.0265153 (PMC8986095; doi:10.1371/journal.pone.0265153)
Supplement: S2 Table — (DOC) [file pone.0265153.s002.doc]

**S2 Table**: Comparison of characteristics of patients with alcohol-related cirrhosis and NAFLD-cirrhosis.

|  | **Alcohol-related cirrhosis**  **(n=53)** | **NAFLD-cirrhosis**  **(n=50)** | **p** |
| --- | --- | --- | --- |
| Age (years) | 63 (59-70) | 67 (58-73) | 0.230 |
| Gender (male) | 44 (83) | 23 (46) | **<0.001** |
| Marital Status  Single/married/divorced/widowed (%) | 6/32/10/5 | 4/35/8/3 | 0.760 |
| Education Status  Primary school/high school/bachelor’s or master’s degree (%) | 19/24/10 | 17/19/14 | 0.529 |
| Diabetes Mellitus (%) | 18 (34) | 33 (66) | **0.001** |
| Arterial hypertension (%) | 17 (32) | 29 (58) | **0.008** |
| Obesity (%) | 8 (15) | 26 (52) | **<0.001** |
| BMI (kg/m^2^) | 27 (23-29) | 30 (28 – 33) | **<0.001** |
| Time since diagnosis (years) | 6.6 (2.7-9.7) | 3.3 (1.6 – 5.3) | **0.002** |
| Hepatic Encephalopathy (%) | 19 (36) | 6 (12) | **0.006** |
| Gastrointestinal bleeding (%) | 12 (23) | 6 (12) | 0.184 |
| Ascites (%) | 32 (60) | 12 (24) | **<0.001** |
| Infections (%) | 8 (15) | 2 (4) | 0.071 |
| AST (IU/L) | 29 (21-42) | 32 (25-47) | 0.116 |
| ALT (IU/L) | 25 (17-38) | 30 (24-42) | **0.014** |
| GGT (IU/L) | 54 (33-88) | 61 (34-90) | 0.794 |
| Bilirubin (mg/dL) | 0.9 (0.6-1.4) | 0.8 (0.6-1.1) | 0.164 |
| Albumin (g/L) | 44 (39-46) | 44 (40-46) | 0.351 |
| INR | 1.1 (1.0-1.3) | 1.1 (1.0-1.2) | 0.382 |
| MELD | 9 (7-12) | 8.0 (6.9 – 10.5) | 0.113 |
| Child-Pugh | 5 (5-7) | 5 (5 – 6) | 0.196 |
| Liver stiffness (kPa) ** | 17 (7.7-26.3) | 19.0 (10.6-26.5) | 0.767 |
| CAP (dB/m) ** | 261 (191-284) | 284 (231-319) | **0.019** |

* Values are numbers or percentages (in brackets) or medians (IQR).

** Available in 41 patients with NAFLD-cirrhosis and 36 patients with alcohol-related cirrhosis within the previous 12 months before the inclusion in the study.
