# Supplementary material for: Stigmatization is common in patients with non-alcoholic fatty liver disease and correlates with quality of life
Source: PLoS One. 2022 Apr 6;17(4):e0265153. doi: 10.1371/journal.pone.0265153 (PMC8986095; doi:10.1371/journal.pone.0265153)
Supplement: S3 Table — (DOC) [file pone.0265153.s003.doc]

**S3 Table**: Stigma-related questions that were answered affirmatively by less than 5% of patients with NAFLD cirrhosis, classified according to the different domains.

| **QUESTIONS** |  |
| --- | --- |
| **STEREOTYPES** | |
| I feel like some people are concerned that my liver disease could be contagious | 3 (2%) |
| I feel like other people think I am a bad person because I have liver disease. | 5 (4%) |
| **DISCRIMIATION** | |
| I feel I have been treated with less respect by others because of my liver disease | 5 (4%) |
| **SHAME** | |
| Because of my liver disease, I sometimes feel useless | 6 (4%) |
| **SOCIAL ISOLATION** | |
| I feel like I am an outsider because of my liver disease | 3 (2%) |
| I avoid doing some things in public because of my liver disease | 3 (2%) |
| Some people avoid me because of my liver disease | 0 (0%) |
| I feel abandoned by family members because of my liver disease. | 1 (1%) |
