# Supplementary material for: Stigmatization is common in patients with non-alcoholic fatty liver disease and correlates with quality of life
Source: PLoS One. 2022 Apr 6;17(4):e0265153. doi: 10.1371/journal.pone.0265153 (PMC8986095; doi:10.1371/journal.pone.0265153)
Supplement: S4 Table — (DOC) [file pone.0265153.s004.doc]

**S4 Table:** Chronic liver diseases questionnaire (CLDQ) scores of the different domains in patients with NAFLD-cirrhosis and patients with alcohol-related cirrhosis.

|  | **Alcohol-related Cirrhosis**  **(n=53)** | **NAFLD-cirrhosis (n=50)** | **p** |
| --- | --- | --- | --- |
| **Abdominal symptoms** | 6.8 (5.8 - 7) | 6 (4.7 - 7) | **0.010** |
| **Fatigue** | 5.6 (4.6 - 6.8) | 5.2 (4 - 6) | 0.165 |
| **Systemic symptoms** | 5.7 (4.8 - 6.6) | 5.6 (4.4 - 6.4) | 0.356 |
| **Activity** | 6.0 (4.7 - 7) | 5 (3.3 - 6) | **0.014** |
| **Emotional function** | 5.9 (5.1 - 6.3) | 5.1 (3.9 - 6.4) | 0.120 |
| **Worry** | 6.2 (5 - 7) | 5.8 (4.2 - 6.8) | 0.205 |
| **TOTAL** | 5.8 (5.1 - 6.4) | 5.5 (4 – 6.1) | **0.045** |

Values are medians and IQR (in brackets)
